# Treatment of central precocious puberty using low dose GnRH analogs (GnRHa)

**DOI:** 10.1186/1687-9856-2015-S1-P89

**Published:** 2015-04-28

**Authors:** Rahul Jahagirdar, Vaman Khadilkar, Ruma Deshpande

**Affiliations:** 1 Bharati Vidyapeeth University Medical College, Pune, Maharashtra, India

## Aims

1. To study the efficacy of lower dose GnRHa in pubertal suppression and bone age advancement.

2. To study if low dose of GnRHa avoided the need for concomitant use of GH therapy while causing adequate pubertal suppression.

## Methods

Clinical Records of 17 children (16 girls and 1 boy) were retrospectively analyzed over a period of 24 months (Mar 2012 to Mar 2014).

All children satisfied the criteria for precocity based on clinical features, radiological assessment and GnRHa stimulation test. All 16 girls had idiopathic precocious puberty and the only boy had hypothalamic hamartoma.

The mean age of presentation was 6. 9 years.

All children received GnRHa as monthly injection in an average dose of 190 µgm /kg/month and were regularly followed for evaluation of pubertal suppression, height gain and Bone age advancement.

## Results

Over a 24 month study period, children showed good pubertal suppression with a comparatively low dose of GnRHa (190 µgm/kg/month). Mean LH post stimulation while on therapy was 2.31 miu/ml suggesting adequate suppression, average annual growth velocity was 5.1 cm. Advancement in bone age was 1.4 years over a period of 2 years.

## Conclusion

1. Children with CPP could be managed with a lower dose of GnRHa to achieve adequate pubertal suppression (190 µgm/kg/month as opposed to the standard recommended dose of 300 - 750 µgm/kg/month).

2. While adequately suppressing puberty and controlling bone age advancement, lower dose of GnRHa seems to allow normal height gain thus negating the need for concomitant GH therapy in an economically challenging situation.

